# Fumonisin B_1_ Induces Immunotoxicity and Apoptosis of Chicken Splenic Lymphocytes

**DOI:** 10.3389/fvets.2022.898121

**Published:** 2022-05-24

**Authors:** Fenghua Zhu, Yang Wang

**Affiliations:** College of Animal Science and Technology, Qingdao Agricultural University, Qingdao, China

**Keywords:** fumonisin B_1_, chicken, splenic lymphocytes, immunotoxicity, apoptosis

## Abstract

Fumonisin B_1_ (FB_1_), produced by *Fusarium*, is among the most abundant and toxic mycotoxin contaminations in feed, causing damages to the health of livestock. However, the mechanisms of FB_1_ toxicity in chickens are less understood. As splenic lymphocytes play important roles in the immune system, the aim of this study was to investigate the immunotoxic effects and mechanisms of FB_1_ on chicken splenic lymphocytes. In the present study, the chicken primary splenic lymphocytes were harvested and treated with 0, 2.5, 5, 10, 20 and 40 μg/mL FB_1_. Then, the cell proliferation, damage, ultrastructure, inflammation and apoptosis were evaluated. Results showed that the proliferation rate of splenic lymphocytes was decreased by FB_1_ treatments. The activity of lactate dehydrogenase (LDH) was increased by FB_1_ treatments in a dose-dependent manner, implying the induction of cell damage. Consistently, the ultrastructure of splenic lymphocytes showed that FB_1_ at all the tested concentrations caused cell structure alterations, including nuclear vacuolation, mitochondrial swelling and mitochondrial crest fracture. Besides, immunosuppressive effects of FB_1_ were observed by the decreased concentrations of interleukin-2 (IL-2), IL-4, IL-12 and interferon-γ (IFN-γ) in the cell culture supernatant. Furthermore, apoptosis was observed in FB_1_-treated cells by flow cytometry. The mRNA expressions of apoptosis-related genes showed that the expression of *Bcl-2* was decreased, while the expressions of the *P53, Bax, Bak-1*, and *Caspase-3* were increased with FB_1_ treatment. Similar results were found in the concentrations of apoptosis-related proteins in the cell supernatant by ELISA assay. Moreover, regression analysis indicated that increasing FB_1_ concentration increased LDH activity, concentrations of Bax, Bak-1 and mRNA expression of *Bak-1* linearly, increased M1 area percentage quadratically, decreased concentration of IFN-γ, mRNA expression of *Bcl-2* linearly, and decreased concentrations of IL-2 and IL-4 quadratically. Besides, regression analysis also showed reciprocal relationships between IL-12 concentration, *Caspase-3* mRNA expression and increasing FB_1_ concentration. The increasing FB_1_ concentration could decrease IL-12 concentration and increase *Caspase-3* mRNA expression. Altogether, this study reported that FB_1_ induced the immunotoxicity of chicken splenic lymphocytes and caused splenic lymphocytes apoptosis by the Bcl-2 family-mediated mitochondrial pathway of caspase activation.

## Introduction

Mycotoxin fumonisins (FBs) are the secondary metabolites produced by *Fusarium verticillioides* and *Fusarium proliferatum* (1). Among the fumonisin homologs, fumonisin B_1_ (FB_1_) is the most prevalent and abundant mycotoxin contamination in stale corn. It is reported that FB_1_ has great potential health hazards to humans and animals (2, 3). FB_1_ can lead to intestinal damage (4, 5), neurotoxity (6, 7) and various cancers (8–10). Moreover, in chickens, FB_1_ exposure lead to reduced performance, nutrient digestibility, immune function, and increased diarrhea as well as mortality (11–14).

The underlying cellular mechanisms of FB_1_-induced toxicity include the induction of oxidative stress, apoptosis and immunotoxicity (15, 16). The immunotoxicity of FB_1_ in chickens may partially be due to the impairment of lymphatic organs and lymphocyte (17, 18). Spleen, a secondary lymphoid organ, is the main filter for blood-borne pathogens and antigens, playing an important role in maintaining immune homeostasis. Circulating T and B cells often gain access to secondary lymphoid organs to search for their cognate antigens (19). Harmful substances, such as cadmium and atrazine can impair chicken spleens (20, 21). It is also reported that exposure to FB_1_ reduced basal rate of splenic lymphocyte proliferation in female mice (22). In chickens, although Todorova et al. showed that FB_1_ affected the immune function by damaging the ultrastructure of splenic lymphocyte (18), the detailed immunotoxicity and molecular mechanisms is still unclear. Thus, in this study, we investigated the immunotoxicity of FB_1_ on splenic lymphocytes by evaluating the proliferation rate, cell damage, the expressions of cytokines, and further tried to examine the potential molecular mechanisms related to apoptosis to provide reference for further research on the toxicity of FB_1_.

## Materials and Methods

### Materials

FB_1_ and RPMI 1640 medium were purchased from Sigma-Aldrich (St. Louis, Mo, USA). Fetal bovine serum (FBS) was purchased from Sijiqing Biological Engineering Materials Co. Ltd. (Hangzhou, China). The methyl thiazolyl tetrazolium (MTT) and lactate dehydrogenase (LDH) assay kits were obtained from Nanjing Jiancheng Bioengineering Institute (Nanjing, China). The chicken interleukin-2 (IL-2), IL-4, IL-6, IL-12, and interferon-γ (IFN-γ) ELISA kits were obtained from Nanjing Jiancheng Bioengineering Institute (Nanjing, China). The propidium iodide (PI) apoptosis detection kit was purchased from BD Pharmingen (Lexington, KY, USA). The chicken B cell CLL/lymphoma-2 (Bc1-2), P53, Bcl-2 associated X (Bax), Bcl-2 antagonist/killer 1 (Bak-1), and cysteinyl aspartate specific proteinase-3 (Caspase-3) ELISA kits were obtained from Qiyi Biological Technology Co. Ltd. (Shanghai, China). RNA extraction kit and the SYBR PremixScript RT-PCR Kit II were purchased from Takara (Shiga, Japan). All other reagents used were of analytical grade.

### Cell Culture and Treatment

The 40-day-old healthy male specific pathogen free (SPF) White Leghorn chickens were obtained from Shandong Academy of Agricultural Sciences for splenic lymphocytes isolation and culture. The use of animals was approved and performed in accordance with the guidelines of Ethics and Animal Welfare Committee of Qingdao Agricultural University. Chickens were given intramuscular injections of ketamine-846 anesthesia mixture (Shengda Animal Medicine Co., Ltd., Dunhua, China) prior to splenic lymphocytes harvesting. Chicken splenic lymphocytes were prepared and cultured according to previous method (23). Briefly, spleen samples were removed from the chickens, washed with sterile cooled phosphate buffered saline (PBS) and ground on ice. The mixture was filtered through a 200-mesh sieve into a Petri dish to collect spleen cell suspension. The lymphocytes were collected by centrifuging at 2,000 *g* for 15 min at room temperature in Histopaque 1077 (Sigma-Aldrich, USA). Then, lymphocytes were washed twice with cooled PBS and re-suspended in RPMI-1640 medium supplemented with 10% FBS, 100 U/mL penicillin, and 100 U/mL streptomycin, and counted using a hemocytometer. Based on trypan blue dye exclusion, when the lymphocytes viability was more than 95%, the cells could be used for the experiments.

In this study, the splenic lymphocytes were treated with 0, 2.5, 5, 10, 20, and 40 μg/mL FB_1_ according to previous studies (24–26), in which 2.5–50 μg/mL FB_1_ suppressed the proliferation of chicken primary cells, such as splenocytes and peripheral lymphocytes. The FB_1_ was dissolved in deionized water to obtain a 40 mg/mL concentration solution. Then, various dilutions of the 40 mg/mL FB_1_ solution were added to cell cultures with final concentrations of 0, 2.5, 5, 10, 20 and 40 μg/mL FB_1_. For MTT assay, splenic lymphocytes were cultured in 96-well microplates (1× 10^4^ cells/mL) under 5% CO_2_ at 42°C, and stimulated with 10 μg/mL concanavalin A (ConA) to induce cell proliferation and treated with 0, 2.5, 5, 10, 20 and 40 μg/mL FB_1_ for 72 h, with 6 parallel holes in each treatment group. For other assays, splenic lymphocytes were cultured in 24-well microplates (1× 10^5^ cells/mL) under 5% CO_2_ at 42°C, and treated with 0, 2.5, 5, 10, 20, and 40 μg/mL FB_1_ for 48 h. Four parallel holes in each treatment group were set for quantitative real-time PCR (RT-PCR) and flow cytometry, and 6 parallel holes were set in each treatment group for the measurement of proliferation rate, inflammatory cytokine levels, LDH activity and apoptosis protein concentrations.

### MTT Assay

After 72 h treatment with ConA and FB_1_, 10 μL MTT solution (5 mg/mL) was added in each well and then incubated for 4 h. After incubated with 100 μL of DMSO, the optical density (OD) was measured at 490 nm using a microplate reader (ThermoFisher MK_3_, USA). Proliferation rate (%) = (OD of cells treated with FB_1_-OD of cells without FB_1_ treatment)/OD of cells without FB_1_ treatment × 100%.

### Transmission Electron Microscopy (TEM)

Cells were fixed in a fresh solution of 0.1 M sodium cacodylate buffer containing 2.5% glutaraldehyde and 2% formaldehyde followed by a 2 h fixation at 4°C with 2% osmium tetroxide in 50 mM sodium cacodylate (pH 7.2). Staining was performed overnight with 0.5% aqueous uranyl acetate. Specimens were dehydrated, embedded in Epon 812 and sectioned into ultrathin slices. The sections were examined on CCD camera system (AMT Corp., USA) (27).

### Analysis of Inflammatory Cytokines and LDH

The concentrations of inflammatory cytokines, including IL-2, IL-4, IL-6, IL-12, IFN-γ and the activity of LDH were determined spectrophotometrically using commercial kits (Nanjing Jiancheng Bioengineering Institute, China) according to manufacturer's protocol.

### Apoptosis Assay by PI Staining

After adding pancreatin (HyClone, USA), the cells were harvested by centrifuging at 1,000 *g*. The cell pellet was fixed in 1.5 mL cold 75% ethanol at 4°C for 8 h. Then, cells were centrifuged, washed in 1 mL PBS and re-suspended in 0.5 mL PBS. To a 0.5 mL cell sample, 0.5 mL RNase A (Sigma-Aldrich, USA) was added, followed by mixing by 1 mL PI (Sigma-Aldrich, USA) solution. The mixed cells were incubated in the dark at room temperature for 30 min and kept at 4°C in the dark until measured. The PI fluorescence was measured using a FC500 flow cytometer (Beckman Coulter, Fullerton, USA).

### RNA Extraction and RT-PCR

Total RNA extraction and reverse transcription were performed according to Mao et al. (28). Primer and Oligo softwares were used for PCR primer sequences (Table 1) design. RT-PCR was performed using Premix Ex TaqTM with SYBR Green (TaKaRa, Dalian, China) and ABI Stepone Real-Time PCR System 7500 Fast Real-Time PCR System (Applied Biosystems, Carlsbad, CA, USA). The thermocycle protocol was 30-s at 95°C followed by 40 cycles of 5-s denaturation at 95°C, 34-s annealing/extension at 60°C, and then a final melting curve analysis to monitor purity of the PCR product. The mRNA abundances of *Bcl-2, Bax, Bak-1, P53* and *Caspase-3* were determined by 2^−ΔΔCq^ method. Relative gene expression concentrations were normalized by eukaryotic reference gene *GAPDH*.

**Table 1:** RT-PCR primer sequences and products.

**Gene**	**Primer sequences (5^′^ → 3^′^)**	**Product size (bp)**	**GeneBank accession No**.
*GAPDH*	TCCTGGTATGACAATGAGTTTGGA	199	NM_204305
	GGGGAGACAGAAGGGAACAGA		
*Bcl-2*	ATCGTCGCCTTCTTCGAGTT	150	Z11961.1
	ATCCCATCCTCCGTTGTCCT		
*Bax*	GTGATGGCATGGGACATAGCTC	90	XM_422067.2
	TGGCGTAGACCTTGCGGATAA		
*Bak-1*	ATGGATGCCTGTCTGTCCTGTTC	106	NM_001030920.1
	GCAGAGCAGTCCAAAGACACTGA		
*P53*	GAGATGCTGAAGGAGATCAATGAG	145	X13057.1
	GTGGTCAGTCCGAGCCTTTT		
*Caspase-3*	ACTCTGGAATTCTGCCTGATGACA	129	NM_204725.1
	CATCTGCATCCGTGCCTGA		

### Analysis of Apoptosis-Related Proteins by ELISA

The concentrations of Bcl-2, Bax, Bak-1, P53 and Caspase-3 in the cell culture supernatant were measured by ELISA kits according to the manufacture instructions (Qiyi Biological Technology Co. Ltd., Shanghai, China). Briefly, standard solutions were prepared. Then, the specimens were thawed at room temperature and 50 μL aliquots were added to the wells covered with a layer of monoclonal antibody, then incubated at 37°C for 30 min together with the standard solutions. Subsequently, the wells were washed and 50 μL of conjugate reagent was added. The wells were further incubated at 37°C for 30 min at the end of which the wells were washed and 100 μL of streptavidin–horseradish peroxidase (HRP)-tagged antibodies were added. The wells were incubated at 37°C for 30 min. Following another round of washing, 100 μL of tetramethylbenzidine was added to the wells. The wells were incubated at room temperature for 30 min in the dark. The reaction was stopped by adding 50 μL stop solution to the wells. The absorbances of the solutions in the wells were measured by a spectrophotometer at 450 nm. A standard curve was plotted from the absorbance values of the standard solutions and the protein concentrations of the samples were calculated from the standard curve.

### Statistical Analysis

One-way ANOVA was performed with the GLM procedure by SAS (SAS Institute Inc, USA, version 9.3), and multiple comparisons were performed with Duncan's test. Results were presented as the means ± standard deviation (SD). The Reg procedure of SAS (SAS Institute Inc, USA, version 9.3) was used for performing the regression of increasing FB_1_ concentration on the measurements. One-dimensional linear equation, one-dimensional quadratic equation, and reciprocal equation were fit. The significant one with the largest *R*^2^ was selected as the most appropriate equation. The differences were considered significant at *P* < 0.05.

## Results

### Effects of Different Concentrations of FB_1_ on the Splenic Lymphocytes Proliferation Rate

**Figure 1:**
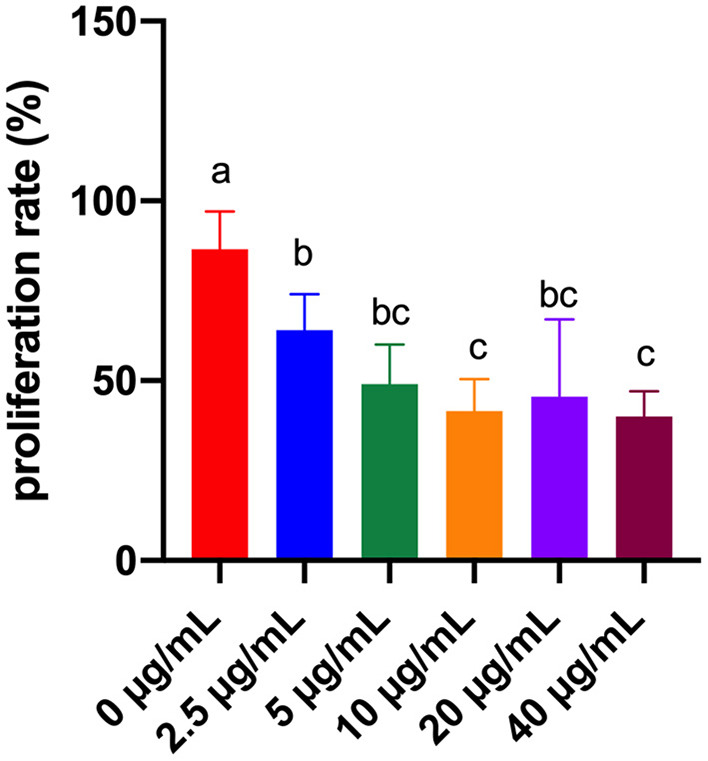
Effects of different concentrations of FB_1_ on the proliferation rate of splenic lymphocytes. Data are presented as the means ± SD for *n* = 6. End-point means without a common letter are significantly different (a, b, or c, *P* < 0.05).

According to Figure 1, compared to the FB_1_-non-treated cells, the lymphocytes proliferation rate was significantly decreased by FB_1_ at 2.5, 5, 10, 20 and 40 μg/mL (*P* < 0.05). Moreover, compared to cells treated with 2.5 μg/mL FB_1_, cells treated with 10 and 40 μg/mL FB_1_ had much lower lymphocytes proliferation rate (*P* < 0.05).

### Effects of Different Concentrations of FB_1_ on the Activity of LDH

**Figure 2:**
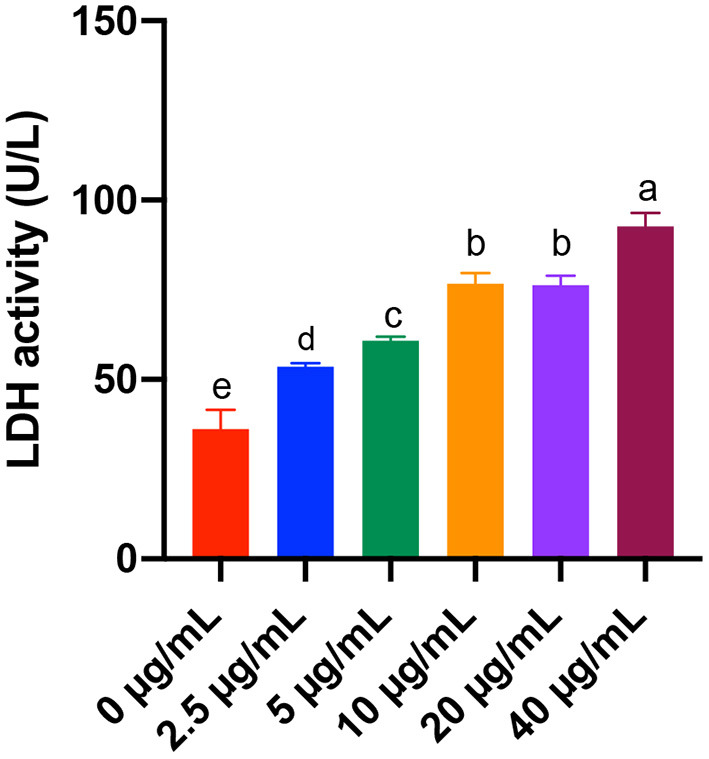
Effects of different concentrations of FB_1_ on the LDH activity in the culture supernatant of splenic lymphocytes. Data are presented as the means ± SD for *n* = 6. End-point means without a common letter are significantly different (a, b, c, d, or e, *P* < 0.05).

As for the LDH, we found that 2.5, 5, 10, 20, and 40 μg/mL FB_1_ significantly increased the LDH activity (*P* < 0.05) in a dose-dependent manner, although there was no significant difference between the 10 and 20 μg/mL FB_1_ treatment groups (*P* > 0.05) (Figure 2).

### Effects of Different Concentrations of FB_1_ on the Ultrastructure of Splenic Lymphocytes

**Figure 3:**
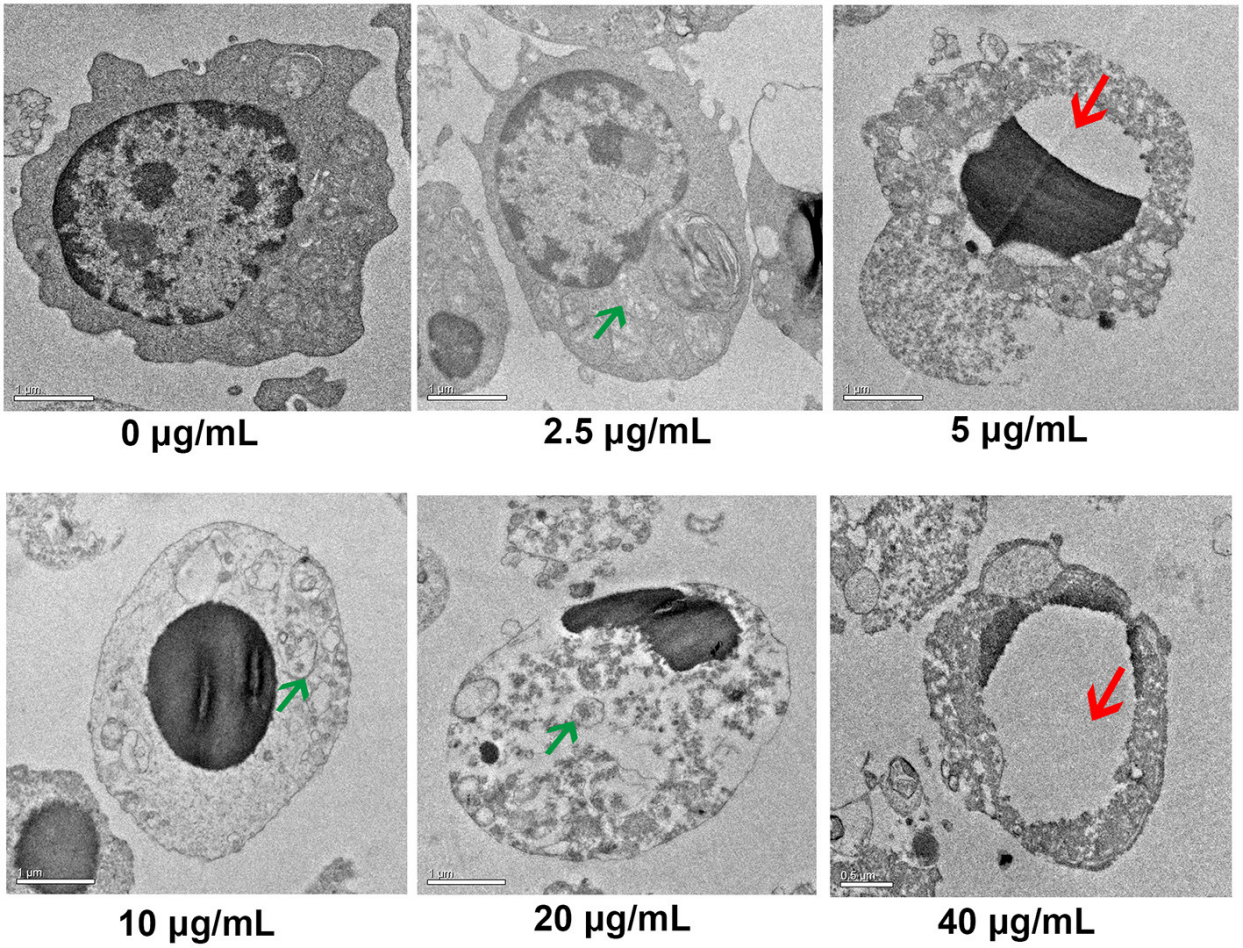
Ultrastructure of splenic lymphocytes by TEM. Green arrows indicate the mitochondrial swelling and mitochondrial crest fracture. Red arrows indicate nuclear vacuolation.

TEM results indicated that the non-treated cells had intact cell membrane and nucleus envelope. Moreover, mitochondria also had intact structure and were not swollen. However, 2.5, 5, 10, 20, and 40 μg/mL FB_1_ caused cell structure damage, including nuclear vacuolation, mitochondrial swelling and mitochondrial crest fracture (Figure 3).

### Effects of Different Concentrations of FB_1_ on the Concentrations of Inflammatory Cytokines

With the treatments of different concentrations of FB_1_, the concentration of IL-2 was significantly decreased compared to the non-treated group (*P* < 0.05). Moreover, a dose-dependent effects of FB_1_ were observed for the reduction of IL-2 concentration. In addition, 5, 10, 20, and 40 μg/mL FB_1_ also significantly down-regulated the concentration of IL-4 in a dose-dependent manner compared to the non-treated and 2.5 μg/mL FB_1_-treated cells (*P* < 0.05). Furthermore, the concentration of IL-12 was also significantly reduced by 5, 10, 20, and 40 μg/mL FB_1_ (*P* < 0.05). Compared to 5 and 10 μg/mL FB_1_, 40 μg/mL FB_1_ treatment led to a much lower IL-12 concentration (*P* < 0.05). Besides, the concentration of IFN-γ was significantly reduced by the FB_1_ at all the tested concentrations (*P* < 0.05), and 40 μg/mL FB_1_ induced the lowest IFN-γ concentration compared to that of other FB_1_ treatments (*P* < 0.05). However, the concentration of IL-6 was not significantly affected by the treatments of FB_1_ (*P* > 0.05) (Table 2).

**Table 2:** Effects of different concentrations of FB_1_ on the inflammatory cytokines.

**FB_**1**_ concentrations (μg/mL)**	**IL-2 (ng/L)**	**IL-4 (ng/L)**	**IL-6 (ng/L)**	**IL-12 (ng/L)**	**IFN-γ (ng/L)**
0	107.12 ± 2.13^a^	123.37 ± 14.24^a^	38.35 ± 1.63	140.24 ± 37.59^a^	84.73 ± 2.84^a^
2.5	95.25 ± 0.64^b^	116.77 ± 2.20^a^	36.02 ± 4.39	123.52 ± 20.52^a^	73.48 ± 7.15^b^
5	87.04 ± 4.62^c^	105.31 ± 3.59^b^	37.30 ± 5.58	92.17 ± 8.43^b^	77.35 ± 3.13^b^
10	51.96 ± 10.16^d^	93.91 ± 4.21^c^	37.58 ± 2.87	91.10 ± 6.61^b^	73.67 ± 2.05^b^
20	37.76 ± 5.12^e^	79.60 ± 4.12^d^	35.23 ± 5.73	76.39 ± 3.01^bc^	72.66 ± 5.30^b^
40	34.54 ± 0.82^e^	62.47 ± 2.83^e^	37.02 ± 1.03	62.01 ± 2.43^c^	65.05 ± 2.44^c^

*Data are presented as the means ± SD for n = 6*.

*a, b, c, d, e Mean value within a row with no common superscript differ significantly (P < 0.05)*.

### Effects of Different Concentrations of FB_1_ on the Apoptosis of Splenic Lymphocytes

**Figure 4:**
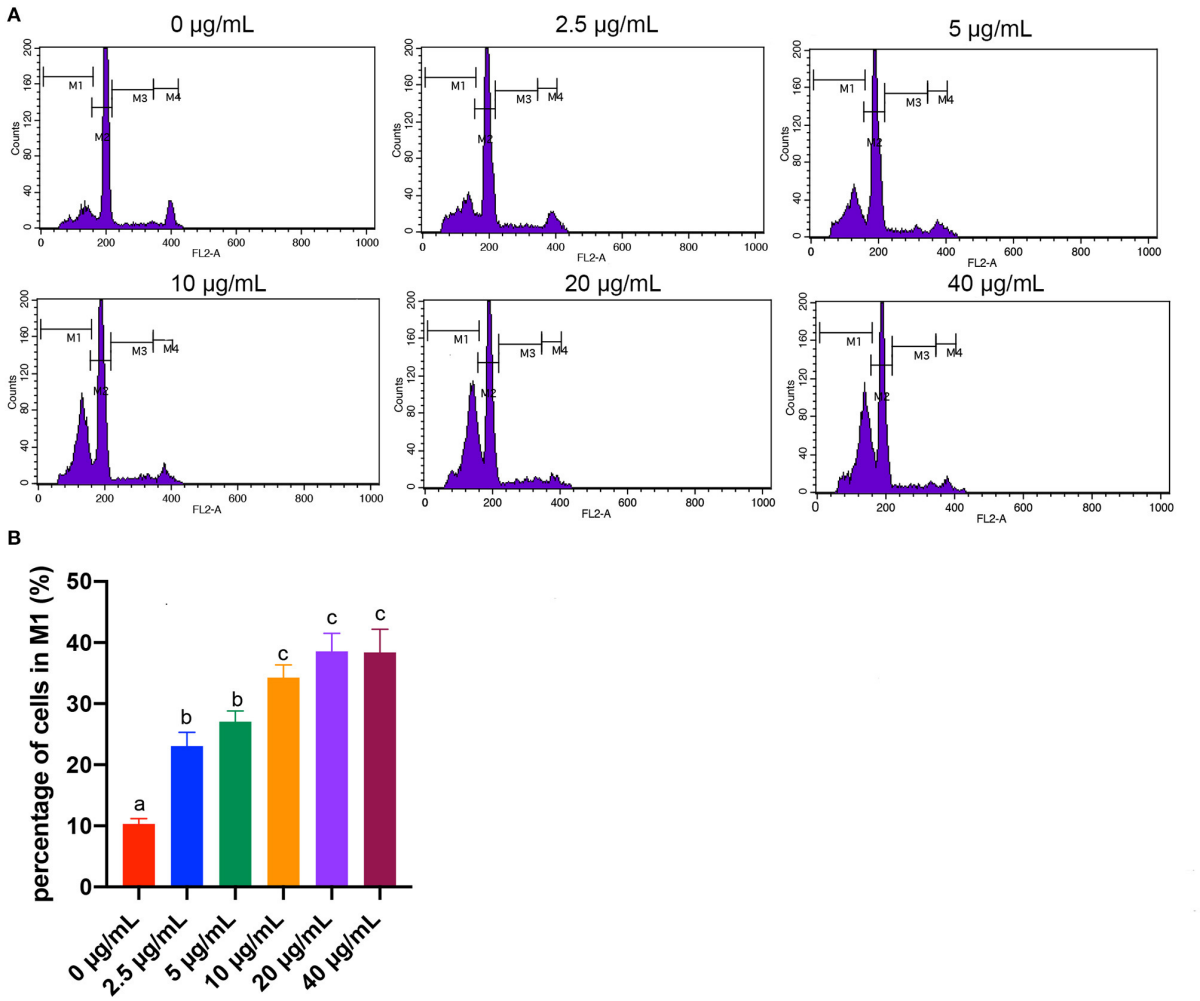
Effects of different concentrations of FB_1_ on the apoptosis of splenic lymphocytes. **(A)** Cell apoptosis was analyzed by flow cytometry. M1 gate indicates the pre-G0/G1 (apoptotic) population, M2 gate indicates the G0/G1 phase, M3 gate indicates the S phase and M4 gate indicates the G2/M phase. **(B)** Percentage of cells in M1 gate. Data are presented as the means ± SD for *n* = 4. End-point means without a common letter are significantly different (a, b, or c, *P* < 0.05).

Apoptotic cells were quantified by flow cytometry analysis. Early apoptotic cells appear in the cell cycle distribution as cells with a hypodiploid DNA. This alteration in DNA content results from degradation of cellular DNA by activation of endogenous endonucleases during apoptosis (29). Thus, cells in the pre-G0/G1 phase (M1) were therefore defined as apoptotic cells (Figure 4). Results showed that the percentages of cells in M1 area of all the FB_1_ treatment groups were significantly increased compared to the non-treated ones (*P* < 0.05). Moreover, compared to the 2.5 and 5 μg/mL FB_1_ treatments, 10, 20, and 40 μg/mL FB_1_ treatments significantly increased the percentages of cells in M1 (*P* < 0.05).

### Effects of Different Concentrations of FB_1_ on the mRNA Expressions of Apoptosis-Related Genes

The expression of *Bcl-2* was significantly down-regulated by 5, 20, and 40 μg/mL FB_1_ compared to the cells treated with 0, 2.5 and 10 μg/mL FB_1_ (*P* < 0.05). And 40 μg/mL FB_1_ led to the lowest *Bcl-2* mRNA expression (*P* < 0.05). The expression of *P53* was only up-regulated in the 40 μg/mL FB_1_ treatment group (*P* < 0.05). Besides, the expression of *Bax* was significantly up-regulated by 10 and 40 μg/mL FB_1_ compared to other groups (*P* < 0.05). In addition, 10, 20 and 40 μg/mL FB_1_ significantly elevated the expression of *Bak-1* (*P* < 0.05). And 40 μg/mL FB_1_ induced the highest expression of *Bak-1* among groups (*P* < 0.05). Furthermore, 10 and 40 μg/mL FB_1_ significantly up-regulated the *Caspase-3* expression compared to the non-treated cells (*P* < 0.05) (Table 3).

**Table 3:** Effects of different concentrations of FB_1_ on the relative expression of apoptosis-related genes.

**FB_**1**_ concentrations (μg/mL)**	***Bcl-2***	***P53***	***Bax***	***Bak-1***	***Caspase-3***
0	1.01 ± 0.06^a^	1.01 ± 0.12^b^	1.03 ± 0.03^bc^	1.01 ± 0.08^c^	1.03 ± 0.18^b^
2.5	0.96 ± 0.04^ab^	1.09 ± 0.11^ab^	0.98 ± 0.04^c^	1.09 ± 0.12^bc^	1.18 ± 0.31^ab^
5	0.86 ± 0.07^b^	1.05 ± 0.10^b^	1.09 ± 0.04^b^	1.27 ± 0.18^b^	1.36 ± 0.29^ab^
10	0.91 ± 0.09^ab^	1.14 ± 0.08^ab^	1.17 ± 0.07^a^	1.19 ± 0.22^bc^	1.65 ± 0.21^a^
20	0.66 ± 0.10^c^	1.01 ± 0.02^b^	1.07 ± 0.02^b^	1.31 ± 0.03^b^	1.52 ± 0.45^ab^
40	0.54 ± 0.02^d^	1.24 ± 0.13^a^	1.22 ± 0.04^a^	1.54 ± 0.10^a^	1.70 ± 0.57^a^

*Data are presented as the means ± SD for n = 4. mRNA expression was standardized to GAPDH expression*.

*^a, b, c^Mean value within a row with no common superscript differ significantly (P < 0.05)*.

### Effects of Different Concentrations of FB_1_ on the Concentrations of Apoptosis-Related Proteins

ELISA results showed that the concentration of Bcl-2 in the cell supernatant was significantly decreased in 5 and 40 μg/mL FB_1_ treatment groups compared to the non-treated group (*P* < 0.05). But there was no significant difference for the Bcl-2 concentration among cells treated with FB_1_ (*P* > 0.05). Moreover, the concentration of P53 was significantly elevated by FB_1_ at all the tested concentrations compared to the non-treated group (*P* < 0.05), in addition, P53 concentration was much higher in 20 and 40 μg/mL FB_1_-treated groups than that of the 2.5 and 5 μg/mL FB_1_-treated groups (*P* < 0.05). FB_1_ at all the tested concentrations also significantly increased the Bax concentration (*P* < 0.05), and 40 μg/mL FB_1_ induced the highest Bax concentration among the groups. Besides, the Bak-1 concentration in the 20 and 40 μg/mL FB_1_-treated groups was much higher than that of the other groups (*P* < 0.05). Furthermore, 40 μg/mL FB_1_ significantly increased Caspase-3 concentration compared to other groups (*P* < 0.05) (Table 4).

**Table 4:** Effects of different concentrations of FB_1_ on the apoptosis-related proteins.

**FB_**1**_ concentrations (μg/mL)**	**Bcl-2 (μg/mL)**	**P53 (μg/mL)**	**Bax (μg/mL)**	**Bak-1 (μg/mL)**	**Caspase-3 (μg/mL)**
0	2.50 ± 0.13^a^	281.75 ± 10.68^c^	3.45 ± 0.17^e^	15.07 ± 1.19^b^	1.58 ± 0.15^bc^
2.5	2.39 ± 0.04^ab^	336.34 ± 29.56^b^	3.74 ± 0.19^d^	13.98 ± 0.98^b^	1.70 ± 0.14^b^
5	2.21 ± 0.23^b^	379.58 ± 28.42^b^	3.91 ± 0.14^cd^	14.29 ± 1.09^b^	1.51 ± 0.12^c^
10	2.35 ± 0.09^ab^	340.76 ± 30.42^b^	4.23 ± 0.20^b^	14.55 ± 0.96^b^	1.72 ± 0.14^b^
20	2.35 ± 0.22^ab^	469.84 ± 17.63^a^	4.11 ± 0.19^bc^	17.48 ± 0.40^a^	1.48 ± 0.05^c^
40	2.16 ± 0.23^b^	445.86 ± 55.15^a^	5.12 ± 0.29^a^	17.52 ± 1.57^a^	1.94 ± 0.13^a^

*Data are presented as the means ± SD for n = 6*.

*^a, b, c, d^Mean value within a row with no common superscript differ significantly (P < 0.05)*.

### Regression Analysis

Regression equations are presented in Table 5. Results indicated that increasing FB_1_ concentration increased the activity of LDH, the concentrations of Bax, Bak-1 and mRNA expression of *Bak-1* linearly, increased percentage of M1 area quadratically, decreased concentration of IFN-γ, mRNA expression of *Bcl-2* linearly, and decreased concentrations of IL-2 and IL-4 quadratically. Rregression analysis also showed reciprocal relationship between IL-12 concentration, *Caspase-3* mRNA expression and increasing FB_1_ concentration. The increasing FB_1_ concentration could decrease IL-12 concentration and increase *Caspase-3* expression. Proliferation rate tended to decrease with increasing FB_1_ concentration (*P* = 0.06).

**Table 5:** Regression equations for FB_1_ concentrations as a function for biochemical indices of splenic lymphocytes.

**Biochemical indices**	**Equation**	***R* ^2^**	***P-*value**
Proliferation Rate (%)	y = 50.47/x + 42.28	0.74	0.06
IL-2 (ng/L)	y = 0.10x^2^-5.71x + 108.49	0.96	0.04
IL-4 (ng/L)	y = 0.04x^2^-2.87x + 121.02	0.99	0.01
IL-12 (ng/L)	y = 143.20/x + 66.84	0.91	0.01
INF-γ (ng/L)	y = −0.27x + 76.64	0.84	0.03
LDH (U/L)	y = 0.94x + 57.32	0.85	0.03
M1 (%)	y = −0.03x^2^ + 1.50x + 20.24	0.98	0.02
Bax (μg/mL)	y = 0.03x + 3.71	0.90	0.01
Bak-1 (μg/mL)	y = 0.10x + 13.96	0.78	0.05
*Bcl-2*	y = −0.01x + 0.96	0.90	0.01
*Bak-1*	y = 0.01x + 1.12	0.87	0.02
*Caspase-3*	y = −1.37/x + 1.70	0.84	0.03

## Discussion

Fusarium mycotoxins such as FB_1_ are the major contaminants in animal feed and induce subclinical symptoms. The spleen of birds is a central immune organ for the proliferation of T and B cells.

In the present study, we aimed to determine the toxic effects and mechanisms of FB_1_ on splenic lymphocytes. We found the proliferation rate of splenic lymphocytes was significantly decreased by treatments of FB_1_ at 2.5, 5, 10, 20, and 40 μg/mL. Similarly, Johnson and Sharma also reported that FB_1_ exposure was able to reduce the lymphocyte proliferation (22). LDH, a key feature of cells undergoing cellular damage, is a stable cytoplasmic enzyme that is found in all cells (30). In the present study, FB_1_ significantly increased the LDH activity in a dose-dependent manner. Regression

analysis also showed that the LDH activity was increased by increasing FB_1_ concentrations. Consistent with this result, the ultrastructure of FB_1_-treated lymphocytes indicated cell impairments, including the mitochondrial swelling, mitochondrial crest fracture and nuclear vacuolation. Cell ultrastructure changes were also observed in a study by Todorova et al. (31). According to this study, the splenic lymphatic nodules were with pale centers, reduced cell number and contained large undifferentiated lymphoblasts or cells with pycnotic nuclei in chickens consuming FB_1_ and deoxynivalenol.

The immunotoxicity of FB_1_ has been associated with decreased immune responses (32). Here, the concentrations of IL-2, IL-4, IL-12, and IFN-γ in the cell culture supernatant were decreased by FB_1_ treatments, and the reductions of IL-2 and IL-4 were in a dose-dependent manner. However, the concentration of IL-6 was not significantly changed by FB_1_. Regression analysis further demonstrated that the concentrations of IL-2, IL-4, IL-12, and IFN-γ were reduced by increasing FB_1_ concentrations. Studies demonstrated that splenocytes from female mice exposed to FB_1_ had a reduced expression of *IL-2* mRNA (22). *In vitro* treatment of swine lymphocytes with FB_1_ significantly decreased IL-4 production (33). Moreover, the secretions of IL-12 induced by LPS exposure of murine bone marrow-derived dendritic cells were suppressed by FB_1_ in a dose dependent manner (34). The mRNA concentration of *IFN-*γ in macrophages was also reduced by supplementation of FB_1_ in chickens (35). Similar with our results, Bhandari et al. reported that the IL-6 concentration in the spleen of FB_1_-treated mice was not significantly altered (36). Unfortunately, no other studies have shown related data on splenic lymphocytes to serve for comparison with our results.

The decrease of cell proliferation might not only be associated with the FB_1_ direct cytotoxicity effects but also be due to apoptosis of lymphocytes (37). Therefore, the pro-apoptotic role of FB_1_ in splenic lymphocytes was further investigated. Previously, studies indicated that FB_1_ induced apoptosis of porcine kidney cells (38), human gastric epithelial cells (39) and turkey peripheral blood lymphocytes (40). Currently, we also observed splenic lymphocytes apoptosis with FB_1_ treatment. Two major apoptotic pathways have been identified: (i) the extrinsic pathway, in which activation of a death receptor by a ligand leads to the activation of initiator Caspase-8; and (ii) the intrinsic pathway, which is caused by cellular stress and cytochrome c release from mitochondria, leading to Caspase-9 activation (41). Once active, Caspase-9 directly cleave and activate Caspase-3 (42). The apoptotic processes that occur before cytochrome c release require a variety of effector molecules, including Bcl-2 family proteins (43, 44). The Bcl-2 family is composed of two subfamilies: one consisting of anti-apoptotic proteins (e.g., Bcl-2, Bcl-XL, Bcl-w, etc.) and the other of pro-apoptotic proteins (e.g., Bax, Bak, Bcl-XS, etc.) (45). In the current study, results of RT-PCR and ELISA demonstrated that the expression of Bcl-2 was decreased, while the expressions of the P53, Bax, Bak-1 and Caspase-3, which can promote apoptosis, were increased with FB_1_ treatment. Regression analysis suggested that the M1 area percentage, concentrations of Bax, Bak-1 and mRNA expressions of *Bak-1* and *Caspase-3* were increased, while the *Bcl-2* mRNA expression was reduced by increasing FB_1_ concentrations. These results indicated that FB_1_ can induce splenic lymphocytes apoptosis by the Bcl-2 family-mediated mitochondrial pathway.

In conclusion, data in this study imply that FB_1_ strongly suppressed the chicken splenic lymphocytes proliferation and caused cell damage, especially the impairment of structure of mitochondria. The immunosuppressive effect of FB_1_ was also found by the decreased concentrations of inflammatory cytokines. Moreover, FB_1_ induced splenic lymphocytes apoptosis through the Bcl-2 family-mediated mitochondrial pathway of caspase activation. Overall, our data provide new evidence for the toxic effects and mechanisms of FB_1_ on chicken and provide new targets for regulating the FB_1_-related subclinical symptoms with possible unfavorable economic outcome.

## Data Availability Statement

The original contributions presented in the study are included in the article/supplementary material, further inquiries can be directed to the corresponding author/s.

## Ethics Statement

The animal study was reviewed and approved by Ethics and Animal Welfare Committee of Qingdao Agricultural University.

## Author Contributions

FZ designed the study. YW analyzed data and wrote the manuscript. FZ and YW performed the research and contributed to revision of the manuscript. Both authors read and approved the final manuscript, contributed to the article and approved the submitted version.

## Funding

This work was supported by the Public Sector Science and Technology Support Program of Qingdao (Grant No. 12-1-3-32-nsh), the National Modern Agricultural Industry Technology System Project of Shandong Province (SDAIT-11-07), National Natural Science Foundation of China (Grant No. 32102586), the Shandong Provincial Natural Science Foundation (Grant No. ZR2020QC183), and the Talents of High Concentration Scientific Research Foundation of Qingdao Agricultural University (Grant No. 663/1119042).

## Conflict of Interest

The authors declare that the research was conducted in the absence of any commercial or financial relationships that could be construed as a potential conflict of interest.

## Publisher's Note

All claims expressed in this article are solely those of the authors and do not necessarily represent those of their affiliated organizations, or those of the publisher, the editors and the reviewers. Any product that may be evaluated in this article, or claim that may be made by its manufacturer, is not guaranteed or endorsed by the publisher.

## References

[1] Lumsangkul C Chiang HI Lo NW Fan YK Ju JC. Developmental toxicity of mycotoxin fumonisin B1 in animal embryogenesis: an overview. Toxins. (2019) 11:114. 10.3390/toxins11020114 30781891 PMC6410136

[2] Schelstraete W Devreese M Croubels S. Comparative toxicokinetics of Fusarium mycotoxins in pigs and humans. Food Chem Toxicol. (2020) 137:111140. 10.1016/j.fct.2020.111140 32004578

[3] Ochieng PE Scippo ML Kemboi DC Croubels S Okoth S Kang'ethe EK. Mycotoxins in poultry feed and feed ingredients from sub-Saharan Africa and their impact on the production of broiler and layer chickens: a review. Toxins. (2021) 13:633. 10.3390/toxins13090633 34564637 PMC8473361

[4] Oswald IP Desautels C Laffitte J Fournout S Peters SY Odin M. Mycotoxin fumonisin B1 increases intestinal colonization by pathogenic *Escherichia coli* in pigs. Appl Environ Microbiol. (2003) 69:5870. 10.1128/AEM.69.10.5870-5874.2003 14532038 PMC201177

[5] Bouhet S Hourcade E Loiseau N Fikry A Martinez S Roselli M. The mycotoxin fumonisin B1 alters the proliferation and the barrier function of porcine intestinal epithelial cells. Toxicol Sci. (2004) 77:165–71. 10.1093/toxsci/kfh006 14600282

[6] Doi K Uetsuka K. Mechanisms of mycotoxin-induced neurotoxicity through oxidative stress-associated pathways. Int J Mol Sci. (2011) 12:5213–37. 10.3390/ijms12085213 21954354 PMC3179161

[7] Domijan AM. Fumonisin B(1): a neurotoxic mycotoxin. Arh Hig Rada Toksikol. (2012) 63:531–44. 10.2478/10004-1254-63-2012-2239 23334049

[8] Wang H Wei H Ma J Luo X. The fumonisin B1 content in corn from North China, a high-risk area of esophageal cancer. J Environ Pathol Toxicol Oncol. (2000) 19:139–41. Available online at: https://europepmc.org/article/med/10905519 10905519

[9] Alizadeh AM Rohandel G Roudbarmohammadi S Roudbary M Sohanaki H Ghiasian SA. Fumonisin B1 contamination of cereals and risk of esophageal cancer in a high risk area in northeastern Iran. Asian Pac J Cancer Prev. (2012) 13:2625–8. 10.7314/APJCP.2012.13.6.2625 22938431

[10] Müller S Dekant W Mally A. Fumonisin B1 and the kidney: modes of action for renal tumor formation by fumonisin B1 in rodents. Food Chem Toxicol. (2012) 50:3833–46. 10.1016/j.fct.2012.06.053 22771819

[11] Sobrane Filho ST Junqueira OM de Laurentiz AC da Silva Filardi da Silva Rubio M Duarte KF. Effects of mycotoxin adsorbents in aflatoxin B 1-and fumonisin B 1-contaminated broiler diet on performance and blood metabolite. R Bras Zootec. (2016) 45:250–256. 10.1590/S1806-92902016000500007

[12] Henry MH Wyatt RD Fletchert OJ. The toxicity of purified fumonisin B1 in broiler chicks. Poult Sci. (2000) 79:1378–84. 10.1093/ps/79.10.1378 11055840

[13] Grenier B Dohnal I Shanmugasundaram R Eicher SD Selvaraj RK Schatzmayr G. Susceptibility of broiler chickens to coccidiosis when fed subclinical doses of deoxynivalenol and fumonisins—special emphasis on the immunological response and the mycotoxin interaction. Toxins. (2016) 8:231. 10.3390/toxins8080231 27472362 PMC4999847

[14] Liu JD Doupovec B Schatzmayr D Murugesan GR Bortoluzzi C Villegas AM. The impact of deoxynivalenol, fumonisins, and their combination on performance, nutrient, and energy digestibility in broiler chickens. Poult Sci. (2020) 99:272–9. 10.3382/ps/pez484 32416811 PMC7587770

[15] Stockmann-Juvala H Savolainen K. A review of the toxic effects and mechanisms of action of fumonisin B1. Hum. Exp. Toxicol. (2008) 27:799–809. 10.1177/0960327108099525 19244287

[16] Bhatti SA Khan MZ Saleemi MK Saqib M Khan A ul-Hassan Z. Protective role of bentonite against aflatoxin B1- and ochratoxin A-induced immunotoxicity in broilers. J Immunotoxicol. (2017) 14:66–76. 10.1080/1547691X.2016.1264503 28094577

[17] Mwanza M Kametler L Bonai A Rajli V Kovacs M Dutton KMF. The cytotoxic effect of fumonisin B1 and ochratoxin A on human and pig lymphocytes using the Methyl Thiazol Tetrazolium (MTT) assay. Mycotoxin Res. (2009) 25:233–8. 10.1007/s12550-009-0033-z 23605153

[18] Todorova KS Kril AI Dimitrov PS Gardeva EG Toshkova RA Tasheva YR. Effect of fumonisin B1 on lymphatic organs in broiler chickens-pathomorphology. Bull Vet Inst Pulawy. (2011) 55:801–5. 10.2376/0005-9366-124-831627116

[19] Bronte V Pittet MJ. The spleen in local and systemic regulation of immunity. Immunity. (2013) 39:806–918. 10.1016/j.immuni.2013.10.010 24238338 PMC3912742

[20] Chen D Zhang Z Yao H Liang Y Xing H Xu S. Effects of atrazine and chlorpyrifos on oxidative stress-induced autophagy in the immune organs of common carp (Cyprinus carpio L.). Fish Shellfish Immun. (2015). 44:12–20. 10.1016/j.fsi.2015.01.014 25652291

[21] Chen M Li X Fan R Yang J Jin X Hamid S. Cadmium induces BNIP3- dependent autophagy in chicken spleen by modulating miR-33-AMPK axis. Chemosphere. (2017) 194:396–402. 10.1016/j.chemosphere.2017.12.026 29223809

[22] Johnson VJ Sharma RP. Gender-dependent immunosuppression following subacute exposure to fumonisin B1. Int Immunopharmacol. (2001) 1:2023–34. 10.1016/S1567-5769(01)00131-X 11606033

[23] Xiao Y Xu S Zhao S Liu K Lu Z Hou Z. Protective effects of selenium against zearalenone-induced apoptosis in chicken spleen lymphocyte via an endoplasmic reticulum stress signaling pathway. Cell Stress Chaperon. (2019) 24:77–89. 10.1007/s12192-018-0943-9 30374880 PMC6363622

[24] Wu W Liu T Vesonder RF. Comparative cytotoxicity of fumonisin B1 and moniliformin in chicken primary cell cultures. Mycopathologia. (1995) 132:111–6. 10.1007/BF01103783 8819833

[25] Li YC Ledoux DR Bermudez AJ Fritsche KL Rottinghaus GE. Effects of fumonisin B1 on selected immune responses in broiler chicks. Poult Sci. (1999) 78:1275–82. 10.1093/ps/78.9.1275 10515357

[26] Keck BB Bodine AB. The effects of fumonisin B1 on viability and mitogenic response of avian immune cells. Poult Sci. (2006) 85:1020–4. 10.1093/ps/85.6.1020 16776470

[27] Hackenberg S Friehs G Kessler M Froelich K Ginzkey C Koehler C. Nanosized titanium dioxide particles do not induce DNA damage in human peripheral blood lymphocytes. Environ Mol Mutagen. (2011) 52:264–8. 10.1002/em.20615 20740634

[28] Mao Y Wang B Xu X Du W Li W Wang Y. Glycyrrhizic acid promotes M1 macrophage polarization in murine bone marrow-derived macrophages associated with the activation of JNK and NF-kappaB. Mediators Inflamm. (2015) 2015:372931. 10.1155/2015/372931 26664149 PMC4668314

[29] Liu KZ Li J Kelsey SM Newland AC Mantsch HH. Quantitative determination of apoptosis on leukemia cells by infrared spectroscopy. Apoptosis. (2001) 6:269–78. 10.1023/A:1011383408381 11445669

[30] Kumar P Nagarajan A Uchil D. Analysis of cell viability by the lactate dehydrogenase assay. Cold Spring Harb Protoc. (2018) 2018:6. 10.1101/pdb.prot095497 29858337

[31] Todorova K Dimitrov P Toshkova R Lazaroza S Gardeva E Yossifova L. Influence of fumonisin B1 and deoxynivalenol on the immune system of chickens after application in quantities, naturally presented in fodders. Medecine. (2014) 67:139–44. Available online at: https://www.researchgate.net/publication/281775009

[32] Li YC Ledoux DR Bermudez AJ Fritsche KL Rottinghaus GE. The individual and combined effects of fumonisin B1 and moniliformin on performance and selected immune parameters in turkey poults. Poult Sci. (2000) 79:871–8. 10.1093/ps/79.6.871 10875770

[33] Taranu I Marin DE Bouhet S Pascale F Bailly JD Miller JD. Mycotoxin fumonisin B1 alters the cytokine profile and decreases the vaccinal antibody titer in pigs. Toxicol Sci. (2005) 84:301–7. 10.1093/toxsci/kfi086 15659571

[34] Li Y Fan Y Xia B Xiao Q Wang Q Sun W. The immunosuppressive characteristics of FB1 by inhibition of maturation and function of BMDCs. Int Immunopharmacol. (2017) 47:206–11. 10.1016/j.intimp.2017.03.031 28432936

[35] Cheng YH Ding ST Chang MH. Effect of fumonisins on macrophage immune functions and gene expression of cytokines in broilers. Arch Anim Nutr. (2006) 60:267–76. di: 10.1080/17450390600785079 10.1080/17450390600785079 16921924

[36] Bhandari N Brown CC Sharma RP. Fumonisin B1-induced localized activation of cytokine network in mouse liver. Food Chem Toxicol. (2002) 40:1483–91. 10.1016/S0278-6915(02)00075-3 12387313

[37] Jones C Ciacci-Zanella JR Zhang Y Henderson G Dickman M. Analysis of fumonisin B1-induced apoptosis. Environ Health Perspect. (2001) 109(Suppl. 2):315–20. 10.1289/ehp.01109s2315 11359701 PMC1240681

[38] Chen J Yang S Huang S Yan R Wang M Chen S. Transcriptome study reveals apoptosis of porcine kidney cells induced by fumonisin B1 via TNF signalling pathway. Food Chem Toxicol. (2020) 139:111274. 10.1016/j.fct.2020.111274 32198028

[39] Yu S Jia B Yang Y Liu N Wu A. Involvement of PERK-CHOP pathway in fumonisin B1-induced cytotoxicity in human gastric epithelial cells. Food Chem Toxicol. (2020) 136:111080. 10.1016/j.fct.2019.111080 31891755

[40] Dombrink-Kurtzman MA. Fumonisin and beauvericin induce apoptosis in turkey peripheral blood lymphocytes. Mycopathologia. (2003) 156:357–64. 10.1023/B:MYCO.0000003607.69016.d2 14682463

[41] Fuchsluger TA Jurkunas U Kazlauskas A Dana R. Corneal endothelial cells are protected from apoptosis by gene therapy. Hum Gene Ther. (2011) 22:549–58. 10.1089/hum.2010.079 21158568 PMC3081440

[42] Brentnall M Rodriguez-Menocal L De Guevara RL Cepero E Boise LH. Caspase-9, caspase-3 and caspase-7 have distinct roles during intrinsic apoptosis. BMC Cell Biol. (2013) 14:32. 10.1186/1471-2121-14-32 23834359 PMC3710246

[43] Scorrano L Korsmeyer SJ. Mechanisms of cytochrome c release by proapoptotic BCL-2 family members. Biochem Biophys Res Commun. (2003) 304:437–44. 10.1016/S0006-291X(03)00615-6 12729577

[44] Burlacu A. Regulation of apoptosis by Bcl-2 family proteins. J Cell MolMed. (2003) 3:249–57. 10.1111/j.1582-4934.2003.tb00225.x 14594549 PMC6741335

[45] Wu X. Study of anti-tumor and immunomodulatory activities of two annonaceous acetogenins: microcarpacin A and microcarpacin B. [dissertation/doctoral thesis]. Hong Kong: Chinese University of Hong Kong.

